# Effectiveness of a patient-centred sleep study report in the management of obstructive sleep apnoea

**DOI:** 10.1007/s11325-022-02573-7

**Published:** 2022-02-09

**Authors:** Meera Srinivasan, Joseph M. Duncan, Michael W. O. Hibbert, David Joffe, Anna M. Mohammadieh, Gary L. Cohen, Peter A. Cistulli, Andrew S. L. Chan

**Affiliations:** 1 Department of Respiratory and Sleep Medicine, Royal North Shore Hospital, St Leonards, Australia; 2 Faculty of Medicine and Health, The University of Sydney, Sydney, Australia

**Keywords:** Obstructive sleep apnoea, Patient-centred care, Education, Adherence, Continuous positive airway pressure

## Abstract

**Purpose:**

Obstructive sleep apnoea (OSA) is a common condition with a range of short- and long-term health implications. Providing patient-centred care is a key principle to ensure patients are well informed and empowered to participate in clinical decision making. This study aimed to develop a patient-centred sleep study report for patients with obstructive sleep apnoea and to determine whether or not its implementation led to improved patient understanding of their disease.

**Methods:**

The study was performed in two phases. The first phase utilised the Delphi-survey technique to develop and critically appraise a patient-centred sleep study report (PCSR) for patients with OSA, to accurately and simply convey key components of the patient’s diagnosis and management. The second phase was a prospective, randomised controlled trial to assess the effect of the PCSR on patient knowledge, self-efficacy, and understanding as measured through validated patient questionnaires.

**Results:**

The PCSR was developed on key concepts deemed to be important by the surveyed physicians, senior sleep scientists and patients. This included ensuring the results were customised, highlighting the patient’s apnoea-hypopnea index, oxygen desaturation index and arousal index and limiting technical information to a few key pieces. Patients randomised to receive the PCSR had improved understanding and perceived patient-physician interaction compared to those randomised to standard care.

**Conclusion:**

The development and implementation of the PCSR was feasible and improved patient understanding and perceived patient-physician interaction in patients with moderate to severe OSA. Whether or not use of the PCSR will translate to improved compliance with therapy will require further evaluation.

## Introduction

Obstructive sleep apnoea (OSA) is a condition characterised by complete or partial obstruction of the upper airway during sleep, resulting in oxygen desaturation or arousal from sleep [1]. It affects up to 38% of the adult population with an increased prevalence in men, obese individuals and in advancing age [2]. Symptoms of OSA include non-restorative sleep, daytime tiredness, impaired memory and concentration and an increased risk of motor vehicle accidents [3–6]. In the long term, severe obstructive sleep apnoea is associated with an increased risk of cardiovascular events [7].

Continuous positive airway pressure (CPAP) provides air pressure to prevent upper airway collapse and reduce obstructive events in sleep. It is highly efficacious in the treatment of OSA; however, its effectiveness is limited by patient adherence to therapy [8, 9]. There is increasing recognition that patient-centred care is an essential foundation for providing high-quality health care and ensuring patient safety. A key principle of this is the provision of information, education and shared knowledge [10]. It is well established in the medical literature that better health outcomes are obtained when patients are empowered to participate in clinical decision making [11].

Standard physician-focused sleep study reports provide complex information that is difficult for patients to interpret and understand. The aim of this study was to develop, implement, and assess the efficacy of a patient-centred sleep study report (PCSR) within the sleep laboratory of a tertiary referral hospital.

The relationship between patient-physician interaction, self-efficacy, health literacy, and health outcomes has been established throughout the medical literature [12–15]. Ellender and colleagues demonstrated that inadequate health literacy was associated with a twofold increased risk of inadequate CPAP usage in a cohort of patients with obstructive sleep apnoea [16]. The relationship between poorer health literacy and worse patient outcomes has also been demonstrated in a range of other health conditions. Provision of written information in addition to verbal communication has also been demonstrated to improve patient care in other health conditions [17]. Strong patient-physician relationships are also positively correlated with improved patient self-efficacy [18].

By developing a PSCR through careful patient and physician engagement, this study aimed to improve understanding, apnoea specific knowledge, and health literacy with the goal of ultimately improving patient-physician interaction and health outcomes.

The preliminary results of this study have been previously reported in the form of abstracts [19, 20].

## Materials and methods

The study was performed in two phases. The first phase involved the development and critical appraisal of the PCSR. The second phase was a prospective, randomised controlled trial to determine if the PSCR had an effect on patient knowledge, self-efficacy and understanding. The study was conducted at the Centre for Sleep Health and Research, Royal North Shore Hospital, a tertiary referral hospital in metropolitan Sydney. The study protocol was approved by the Human Research Ethics Committee of the Northern Sydney Local Health District prior to commencement.

The first phase of the study involved the development of the PSCR using the Delphi-survey technique. Patients and clinicians were recruited from Royal North Shore Hospital staff and sleep clinics. A multidisciplinary panel of sleep clinicians and senior sleep scientists were given background on the goals of the patient-centred sleep study report.

They were provided a questionnaire with a short answer format and asked a series of open-ended questions about the content that they thought should be included in the PSCR. The questions asked respondents the most important parameters required to succinctly convey information about the patient’s sleep study, the most important health implications of obstructive sleep apnoea that should be emphasised to the patient and which treatment options should be discussed. The results from this questionnaire were used to inform a second survey which comprised of two parts. The first was a list of the top eight sleep parameters and the second was the top eight health outcomes as extracted from answers submitted by physicians in the first stage of the process. These lists were provided to clinicians and patients who were asked to rank the relative importance of different components in numerical order. In addition, patients were also asked open-ended questions to assess their baseline understanding and knowledge about sleep disorders and ascertain the additional information they would want to know about their sleep study. The findings of this second survey formed the basis of the PSCR which was refined by physicians, sleep scientists and patient groups to optimise language, layout and ensure readability.

In the second phase of the study, patients with moderate to severe OSA, defined as an apnoea-hypopnoea index of ≥ 15 events per hour on diagnostic polysomnography, were recruited from the sleep laboratory at Royal North Shore Hospital. Apnoeas were defined by a cessation of airflow for at least 10 seconds in association with oxygen desaturation of at least 3% or an arousal. Hypopnoeas were defined by a reduction in the amplitude of airflow as measured using nasal pressure or thoracoabdominal wall movement, by > 50% of the baseline measurement for > 10 seconds, in association with oxygen desaturation of at least 3% or an arousal. Patients were randomised via block randomisation to receive the PCSR or standard care.

The PCSR was incorporated into the compumetics profusion PSG4 sleep study reporting software so that it was automatically generated at the same time as the physician-centred sleep study report. Patients who were randomised to the PSCR received it after their sleep study and before their follow-up consultation with their sleep physician to discuss their results.

Baseline demographic information including age, height, weight, ethnicity and highest education level obtained was collected. The efficacy of the PCSR was assessed using the Perceived Efficacy in Patient-Physician Interactions scale (PEPPI-5), the Apnoea Knowledge Test (AKT) and the Self-Care Management tool (SCM). The PEPPI-5 was originally developed to measure patient self-efficacy in older patients, particularly in regard to the patient’s perceived ability to obtain the relevant medical information to their chief medical concern [21]. It has subsequently been validated in other cohorts and is strongly correlated with perceived health management skills [22]. The AKT was developed by Smith et al. through a multidisciplinary, expert panel of sleep physicians, sleep psychologists and clinical nurse consultants and aims to specifically assess a patient’s knowledge of OSA and treatment. Assessment of the AKT demonstrated readability, with language that should be comprehensible to patients with a grade four reading level and demonstrated good reliability with a Cronbach alpha score of 0.6 [23]. The SCM was developed and validated through the chronic disease self-management programme at Stanford University [24]. It has subsequently been used and validated in other cohorts and conditions [25]. Patient understanding was also assessed using a novel questionnaire focusing on aspects identified as important by the Delphi-survey technique.

Data were analysed using a Student’s *t*-test for parametric variables and Wilcoxon rank-sum test for non-parametric variables. Statistical significance was accepted if *p* < 0.05. Results are expressed as mean ± standard deviation.

## Results

**Fig. 1:**
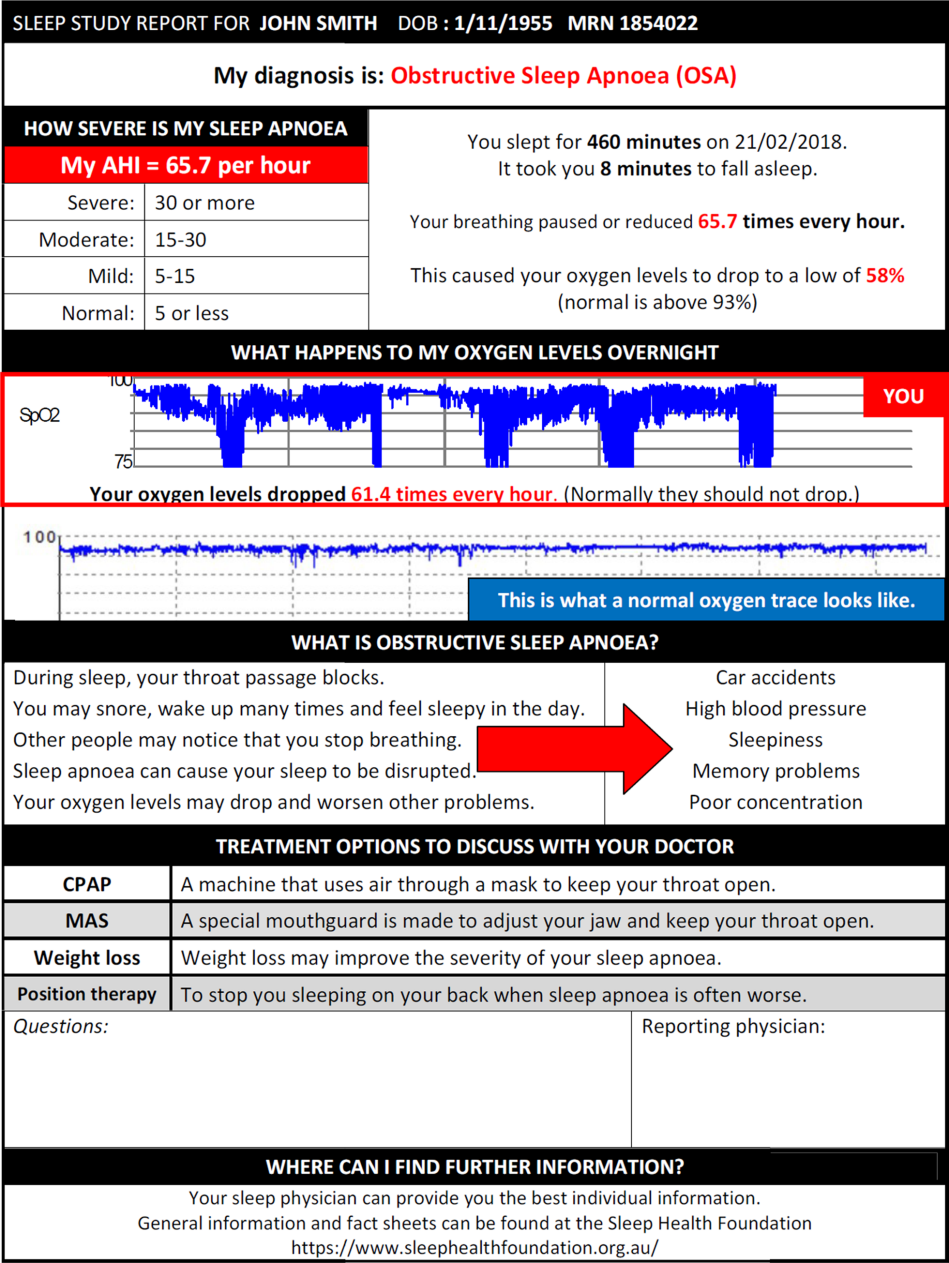
Example of a patient-centred sleep study report

Eight sleep physicians and sleep scientists and 16 patients were involved in the Delphi survey. The results of the Delphi survey identified some common themes that were felt to be important by physicians and patients. Both groups identified the apnoea-hypopnoea index (AHI), the oxygen desaturation index (ODI) and arousal index to be amongst the most important parameters to highlight sleep impairment (see Table 1). Patients ranked sleep efficiency to be the equal third most important parameter while physicians felt the desaturation nadir should be highlighted. These six parameters were all included in the final PSCR (see example provided in Fig. 1).

**Table 1:** Median rank provided by physicians and patients when asked to order the relative importance of sleep parameters in OSA

Most useful for educating patients about their sleep		
	Median rank	
**Item**	Physician	Patient
**AHI**	1.0	1.0
**ODI**	2.0	2.0
**Arousal index**	4.0	3.0
**Sleep stages**	6.0	4.0
**Sleep positions**	5.0	5.5
**SpO2 nadir**	3.0	6.0
**Sleep latency**	8.0	5.0
**Sleep efficiency**	7.0	3.0
***n***	8	16

In terms of the health implications of OSA, both groups emphasised the importance of daytime sleepiness and motor vehicle accidents. The remaining health implications were split evenly by patients while physicians ranked cardiovascular disease and mood disorders above other parameters (see Table 2). These parameters were included in the PSCR.

**Table 2:** Median rank provided by physicians and patients when asked to order the relative importance of health implications in OSA

Most important health implications of OSA		
	Median Rank	
**Item**	Physician	Patient
**Daytime sleepiness**	2.0	1.0
**Atrial fibrillation**	5.5	4.0
**Mood disorders**	4.0	4.5
**Memory impairment**	4.5	4.0
**Cancer risk**	8.0	3
**Reduced life expectancy**	7.0	4
**Cardiovascular disease**	4.0	4
**Motor vehicle accidents**	1.0	3
***n***	8	16

Feedback from the draft PSCR from patients and physicians provided the following suggestions including:

- A maximum of one page summary
- Ensuring the results were customised to the patient
- Including graphics depicting oxygen desaturation

An example of the final PSCR is provided in Fig. 1.

In the implementation phase of the study, 60 patients were recruited with 34 randomised to receive standard care and 26 to receive the PSCR. No statistically significant differences in baseline characteristics were noted between the groups (Table 3). On average, the patients were middle-aged (mean age 56.3 years [± 4.2]) and obese (mean BMI 33.3 [± 0.6]) and had severe OSA (mean AHI 43.8 [± 5.9]).

**Table 3:** Baseline characteristics

Characteristic		Standard care	PCSR	*P*-value
Number		34	26	
Age (mean ± SD)		57.0 (14.4)	55.4 (16.7)	0.69
BMI (mean ± SD)		32.7 (6.5)	34.1 (6.5)	0.41
Ethnicity (%)	Aboriginal/ Torres Strait Island	2.9	3.8	0.99
	Caucasian	64.7	65.4	
	Latino/ Hispanic	2.9	3.8	
	South Asian	5.9	3.8	
	East Asian	8.8	11.5	
	Other	2.9	3.8	
	Mixed	8.8	7.7	
Employment (%)	Full time	38.2	50	0.12
	Part time	11.8	26.9	
	Not employed	11.8	11.5	
	Retired	35.3	11.5	
Education (%)	Year 10	20.6	11.5	0.64
	Year 12	11.8	23.1	
	Certificate/diploma	23.5	30.8	
	Bachelor	26.5	23.1	
	Postgraduate	17.6	11.5	
AHI (mean ± SD)		40.5 (23.5)	48.1 (25.7)	0.24
ODI (mean ± SD)		27.8 (22.9)	36.8 (24.6)	0.15

Patients randomised to receive the PCSR achieved a significantly higher level of self-efficacy, as measured by the PEPPI-5 questionnaire (mean score 20.7 [± 3.6] vs. 18.6[± 3.6] *p* = 0.05) and a significantly higher level of understanding (mean score 15 [± 4] vs. 12.4 [± 2.6] *p* < 0.05). Scores for the Apnoea Knowledge Test (AKT) (mean score 10.4 [± 1.6] vs. 9.5 [± 3.1]) and the Self Care Management tool (31.3 [± 6.6] vs.28.1 [± 7.1]), were numerically higher in the PCSR group although results did not reach statistical significance.

## Discussion

The development and implementation of a PCSR was feasible and improved patient understanding and perceived patient-physician interaction in patients with moderate to severe OSA in this prospective randomised study conducted in a tertiary referral hospital setting.

A Cochrane review assessing the impact of educational interventions to improve CPAP usage in OSA demonstrated that short-term educational interventions resulted in improvement in symptoms and improved CPAP compliance in adults with moderate to severe OSA [26]. The efficacy of patient-centred interventions has previously been demonstrated including in a study by Lin et al. which showed that patients randomised to receive a patient-directed discharge letter had significant improvements in understanding the reasons for their hospitalisation and post-discharge recommendations [27]. Similarly, Mossansen et al. demonstrated that patient-centred pathology reports were associated with greater patient knowledge surrounding a diagnosis of bladder cancer [28]. Promising results were also seen in a pilot study of a patient-centred ultrasound report for hydronephrosis [29]. To our knowledge, this is the first study demonstrating the feasibility and effectiveness of a patient-centred sleep study report in OSA. These results support the wider implementation of the PCSR in the clinical care of patients with OSA. Improving the health literacy of patients with OSA may improve treatment adherence [16].

The lack of difference between the groups for apnoea knowledge and self-care suggests that improvements could be made to the PCSR to ensure that more detailed and specific information is provided to patients. The patients in this study also had higher than average baseline levels of education, with more than half holding tertiary qualifications. This may have reduced the degree of inter-group difference observed.

The patients in this cohort had a relatively high burden of disease at the time of diagnosis with the mean AHI well into the severe range and a high baseline BMI. This may suggest a baseline of poorer self-efficacy and self-care compared to patients with more moderate disease. Cheng et al. (2018) demonstrated in a prospective study that those with a higher BMI had lower levels of baseline health literacy [15]. This may have affected the ability of the PCSR to make an impact in terms of a change to self-care and understanding. The effect of this could be further assessed with an increased sample size and sub-analysis of the effect of the intervention on patients with different degrees of OSA. A baseline measurement of self-efficacy, self-care and knowledge would have also been useful. The inclusion of the PSCR into the sleep reporting software allowed it to be automatically generated for all patients with OSA with no additional cost or workload.

Limitations of the study include the lack of blinding of patients and physicians meaning it is possible that the presence of the PCSR had the consequence of highlighting patient-centred care and focusing the physician–patient discussion. We therefore cannot determine if it was this focused discussion rather than the tool alone that improved patient outcomes. A further study to assess the long-term implications of the PCSR on patient care is planned, including assessing its effect on adherence to CPAP or other therapies for OSA.

## Conclusion

The patient-centred sleep report proved to be a feasible intervention for patients with moderate to severe OSA, cared for in a tertiary referral hospital. The PSCR was developed with patient and physician consultation and was easy to implement in a busy clinical practice. The implementation of the PSCR showed a short-term improvement in patient understanding and perceived interaction with physicians. Longer term analysis and an increased sample size is required to determine if this benefit is sustained and whether or not the improvements can be translated to patients with a milder degree of OSA. Amendments to the PSCR based on the results of this study will be considered to improve apnoea specific knowledge. A future study is planned to assess the effect of the PSCR on adherence to therapy.

## Data Availability

The datasets generated during and/ or analysed during the current study are available from the corresponding author on reasonable request.
